# Deutetrabenazine and Modified Electroconvulsive Therapy for Tardive Dyskinesia With Recurrent Depression: A Case Report

**DOI:** 10.7759/cureus.102749

**Published:** 2026-01-31

**Authors:** Zhiying Wan, Xiaofen Li, Yu Fang, Chao Zeng, Meiyu Shen

**Affiliations:** 1 Department of Psychiatry, Renmin Hospital of Wuhan University, Wuhan, CHN

**Keywords:** anticholinergic drugs, antipsychotic classes, antipsychotics, deutetrabenazine, modified electroconvulsive therapy, recurrent depression, tardive dyskinesia

## Abstract

This study reports a case of tardive dyskinesia (TD) and depression in a patient with long-term use of antipsychotic and anticholinergic drugs, through the tapering and discontinuation of anticholinergic drugs and the concurrent use of deutetrabenazine combined with modified electroconvulsive therapy (MECT). Her TD symptoms have completely improved. The score on the Hamilton Depression Rating Scale (HAMD-17) has dropped from 20 points to eight points, and the depressive symptoms have improved by 60%. A 31-year-old woman with a mood disorder, treated with risperidone (dose range: 4 mL/day, duration: 11 months), olanzapine (dose range: 10 mg/day, duration: 11 months), and benztropine (dose range: 6 mg/day, duration: four months) for recurrent depression, developed lip twitching, bradykinesia, and worsened depression. Upon admission on February 4, benztropine was tapered and discontinued by February 22. A new regimen of deutetrabenazine combined with MECT was initiated on February 7. Following 15 days of this combined treatment, the patient’s TD symptoms completely resolved. The HAMD-17 score dropped from 20 to eight, and the Brief Psychiatric Rating Scale (BPRS) score initially fluctuated but then stabilized and improved. No recurrence was observed during a 10-day follow-up post-discharge. This case study indicates that stopping anticholinergic drugs and using vesicular monoamine transporter 2 (VMAT2) inhibitors with MECT can quickly alleviate symptoms in patients with TD and mood disorders. Clinicians should be aware of the long-term motor disorder risks of antipsychotics, perform regular screenings, and focus on multi-target interventions. Future research should increase sample sizes to confirm the treatment's general applicability and long-term safety.

## Introduction

Tardive dyskinesia (TD) is a movement disorder from prolonged antipsychotic use [1], marked by involuntary, repetitive movements. Its exact cause is unclear, but it may involve dopamine receptor hypersensitivity in the basal ganglia [2]. The therapeutic action of antipsychotics primarily involves antagonism of dopamine D2 receptors. While effective for managing psychosis and certain mood symptoms, chronic blockade of these receptors, particularly within the nigrostriatal pathway of the basal ganglia, is hypothesized to trigger compensatory postsynaptic receptor supersensitivity and maladaptive neuroplastic changes. Mood disorders, such as depression and bipolar disorder, often require adjunctive treatment with antipsychotics [3]. Antipsychotics, particularly second-generation agents, are an important therapeutic option for mood disorders, but their use requires a careful balance between efficacy and the risk of side effects, such as TD. In mood disorders, their primary roles include: serving as augmenting agents in treatment-resistant major depressive disorder, providing rapid stabilization of mood episodes in bipolar disorder, and directly addressing psychotic symptoms in severe depression with psychotic features. However, this therapeutic utility underscores the critical importance of vigilance regarding their long-term risks, such as TD. The co-occurrence of TD and mood disorders may stem from shared neurobiological mechanisms, including alterations in brain structure and neurotransmitter abnormalities [4]. When TD manifests, it significantly complicates the management of mood disorders. There is currently no consensus on treatment strategies, and traditional options like anticholinergic drugs may worsen cognitive issues or mask the symptoms of TD [5]. While second-generation antipsychotics (SGAs) like risperidone and olanzapine have lowered the overall incidence of TD compared to first-generation drugs, a significant risk remains [6]. Notably, TD risk varies among SGAs (e.g., risperidone > clozapine). Thus, even with modern antipsychotics, TD management remains a considerable challenge [7]. A novel targeted approach for TD uses selective vesicular monoamine transporter 2 (VMAT2) inhibitors such as valbenazine and deutetrabenazine. Their promising effect stems from a presynaptic mechanism: by inhibiting VMAT2, they reduce striatal dopamine release, thereby modulating the hyperdopaminergic activity implicated in TD without direct postsynaptic receptor blockade [8]. Modified electroconvulsive therapy (MECT), a well-established biological intervention for severe, treatment-resistant psychiatric conditions, involves the delivery of a controlled electrical stimulus under brief general anesthesia and muscle relaxation to induce a generalized cerebral seizure. Its therapeutic effects in mood disorders are thought to arise from subsequent, widespread neurochemical alterations and modulation of neural network connectivity. This case report discussed a young woman with TD and depression after prolonged antipsychotic and anticholinergic use. Her symptoms improved significantly after stopping anticholinergics and using deutetrabenazine (Austedo) and MECT, offering insights into the comorbidity of TD and mood disorders and suggesting personalized treatment strategies.

## Case presentation

The patient was a 31-year-old unmarried female office worker with a conscientious and responsible pre-morbid personality. She denies any history of trauma, substance abuse, other significant comorbid physical conditions, or family history of mental illness. Her psychological difficulties began in 2017, when she first developed anxiety and symptoms of excessive sensitivity to her surroundings under the pressure of prolonged high-intensity work.

Since then, the patient has been admitted to psychiatric departments twice due to emotional and behavioral disorders: The first hospitalization occurred in October 2023 (lasting four weeks), triggered by her father's death from cardiovascular disease and her mother's diagnosis with a chronic illness. At that time, she presented with symptoms of irritability, depressed mood, anhedonia, fatigue, low self-esteem, reluctance to work, poor sleep, auditory hallucinations involving self-blame, negativity, pessimism, and recurrent suicidal ideation with plans. She was diagnosed with "severe depressive episode with psychotic symptoms." After discharge, she was maintained on long-term oral risperidone and olanzapine (specific doses unknown). The second hospitalization occurred in September 2024 (lasting three weeks), with symptoms largely similar to the previous episode and severe suicidal ideation. A definitive diagnosis of "recurrent depressive disorder, current episode severe with psychotic symptoms" was made. After discharge, she was prescribed oral risperidone, olanzapine, and other medications (details in Table 1), with her condition adequately controlled. On January 9, 2025, the patient presented to our psychiatric outpatient clinic for follow-up due to newly developed symptoms of bradykinesia in all four limbs, slowed thinking, and involuntary mouth opening/closing movements (absent during sleep). She was treated with depakine (valproate), lithium carbonate, benztropine, and lorazepam (details in Table 1), but the aforementioned symptoms showed no significant improvement. For systematic diagnosis and treatment, the patient was admitted to the psychiatric department of our hospital on February 4, 2025, with the admission diagnosis of "mood (affective) disorder."

**Table 1:** Summary of oral medications taken before admission (2023-February 2025) BID, twice a day; DC, discontinue; mg, milligram(s); QD, once daily; QN, every night; TID, three times a day

Oral medication (generic name)	October 2023	September 2024	January 2025	Estimated duration
Risperidone	The specific dosage and frequency are unknown	2 mL BID	DC	11 months
Olanzapine	The specific dosage and frequency are unknown	10 mg QN	DC	11 months
Lithium carbonate	-	300 mg QD; 600 mg QN	300 mg BID	Four months
Lorazepam	-	1 mg QN	1 mg QN	Four months
Benztropine	-	2 mg TID	2 mg TID	Four months
Valproate	-	-	250 mg QN	One month

Upon admission, the patient was in stable condition with vital signs as follows: body temperature 36.6°C, heart rate 96 beats/minute, respiratory rate 18 breaths/minute, and blood pressure 132/86 mmHg. Mental status examination revealed that the patient was conscious, cooperative, fully oriented, engaged appropriately in conversation, and able to answer questions. In terms of cognitive functions, no sensory enhancement or diminution was noted. Gross assessment of intelligence showed no abnormalities, although attention was impaired and memory was poor. Insight was preserved. No hallucinations, delusions, or formal thought disorders were detected. Affect was unstable, characterized by low mood, tension, and worry. Volition and activity were reduced, with observed lip twitching, bradykinesia of the limbs, and interrupted speech due to temporomandibular joint pain. Sleep and appetite were poor. Neurological examination showed no focal positive signs. Cranial nerve examination was normal. Examination of muscle strength, muscle tone, coordination, and sensation in all four limbs revealed no abnormalities. Laboratory tests performed on February 5, 2025, including complete blood count, thyroid function panel (three items), and cardiac enzyme panel (three items), were all within normal limits (Table 2). The sex hormone panel showed elevated testosterone at 66.81 ng/dL, showing an increase [9]. A 12-lead bedside electrocardiogram indicated sinus rhythm with a normal electrical axis. MRI of the brain without contrast showed no significant abnormalities. On February 5, the Brief Psychiatric Rating Scale (BPRS) [10] score was 27, suggesting moderate somatic concern and mild anxiety and tension; the Hamilton Depression Rating Scale (HAMD-17) [11] score was 20, indicating moderate depression (Table 3). The patient was diagnosed with recurrent depressive disorder, TD, and drug-induced parkinsonism.

**Table 2:** Test results after admission

Laboratory test	February 5	Reference range
Testosterone	66.8 ng/dL↑	10.83-56.94 ng/dL
Free triiodothyronine (FT3)	3.29	2.3-4.2 pg/mL
Free thyroxine (FT4）	1.20	0.89-1.76 g/dL
Thyroid-stimulating hormone (TSH）	5.510↑	0.55-4.78 µIU/mL
Creatine kinase-MB (CK-MB)	1.54	0-3.61 ng/mL
Myoglobin	34.6	0-58 µg/L
High-sensitivity troponin T (hs-cTnT)	3.6	0-14 ng/L
Estradiol	62	Follicular phase 21-251 pg/mL; ovulation 38-649 pg/mL; luteal phase 21-312 pg/mL; menopause ≤28 pg/mL
Follicle-stimulating hormone (FSH)	5.81	Follicular phase 3.03-8.08 mIU/mL; ovulation 2.55-16.69 mIU/mL; luteal phase 1.38-5.47 mIU/mL; menopause 26.72-133.41 mIU/mL
Luteinizing hormone (LH)	5.22	Follicular phase 1.8-11.78 mIU/mL; ovulation 7.59-89.08 mIU/mL; luteal phase 0.56-14 mIU/mL; menopause 5.16-61.99 mIU/mL

**Table 3:** The assessment results of the scale after admission

	February 5	February 13	February 20	Reference range
Brief Psychiatric Rating Scale (BPRS)	27 points	29 points	27 points	Mild: total score 31-40; moderate: total score 41-53; severe: total score >53
Hamilton Depression Rating Scale (HAMD)	20 points	Eight points	Not evaluated	No depression (0-7); mild depression (8-16); moderate depression (17-23); severe depression (≥24)

**Figure 1:**
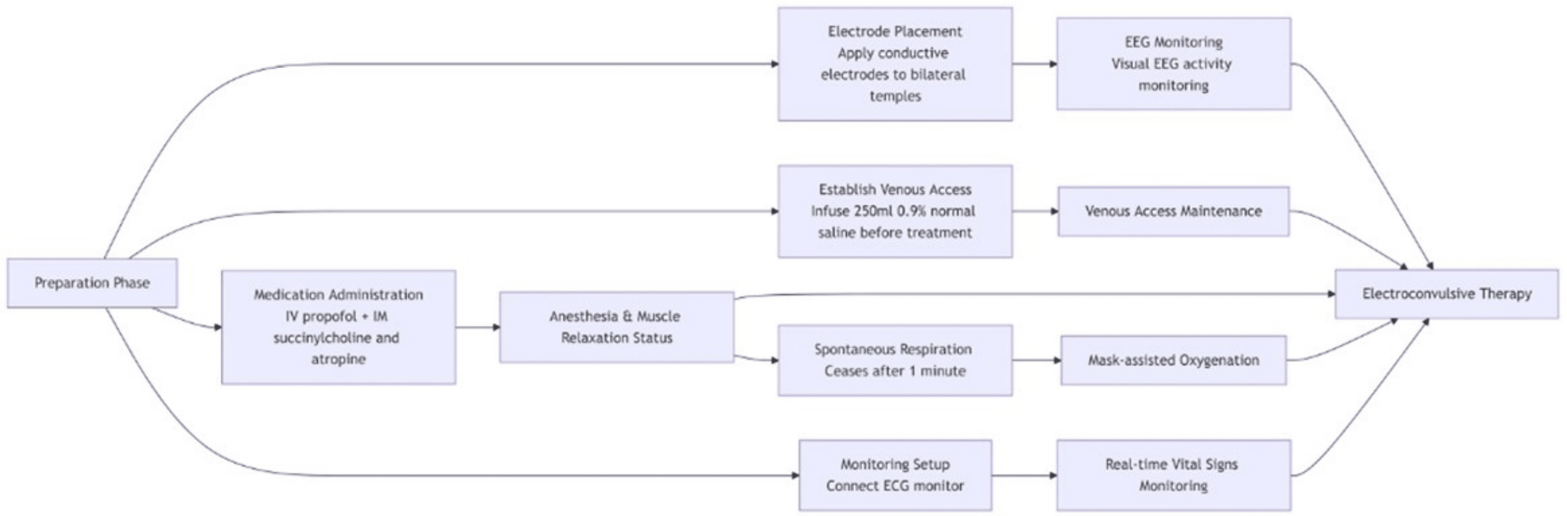
Modified electroconvulsive therapy (MECT) procedure for the patient

The patient's treatment course was as follows: From February 4 to February 6, treatment primarily consisted of oral medications, including benztropine, lithium carbonate, and lorazepam, while other antipsychotic medications were discontinued. On February 7, deutetrabenazine and MECT were added to the existing regimen (details of oral medications and treatments are listed in Table 4). The patient and their family were informed of the relevant precautions for MECT, and the family signed the informed consent form for the procedure. MECT was administered three times per week, totaling seven sessions. The MECT procedure is illustrated in Figure 1. Each session induced an electroencephalographic seizure with moderate amplitude and adequate duration. The patient regained consciousness approximately four minutes after mask oxygen administration on average. By February 10, the patient’s speech function had partially recovered, allowing them to produce sentences of more than 10 words. On February 12, resting tremors had resolved, and jaw pain was alleviated. On February 13, the BPRS score was 29, indicating mild anxiety, somatic concern, and moderate mannerisms and posturing. The HAMD-17 score was eight, indicating mild depression nearing remission. On February 20, the BPRS score was 27, suggesting mild anxiety, depressed mood, and moderate somatic concern. By February 21, the classic triad of oral, lingual, and buccal dyskinesia had completely resolved. MECT was discontinued, and the patient was discharged with a prescription for continued oral deutetrabenazine 6 mg twice daily and lorazepam 1 mg nightly. A telephone follow-up conducted ten days after discharge on March 3 revealed that the patient reported reduced work-related stress, improved mood, enhanced quality of life, and no recurrence of symptoms.

**Table 4:** The situation of oral medication and MECT treatment after admission BID, twice a day; DC, discontinue; mg, milligram(s); MECT, modified electroconvulsive therapy; QD, once daily; QN, every night 20 Hz × 3 s: This notation indicates the amplitude of the spectral component at a center frequency of 20 Hz, calculated from analysis within a three-second time window

	Benztropine	Lithium carbonate	Lorazepam	Deuterobenazine	MECT
Day 1, February 4	2 mg BID	250 mg BID	1 mg BID	-	-
Day 2, February 5	2 mg QD	500 mg BID	1 mg QN	-	-
Day 3, February 6	2 mg QD	500 mg BID	1 mg QN	-	-
Day 4, February 7	2 mg QD	500 mg BID	1 mg QN	6 mg QN	First 20 Hz × 3 s
Day 5, February 8	2 mg QD	500 mg BID	1 mg QN	6 mg QN	-
Day 6, February 9	2 mg QD	500 mg BID	1 mg QN	6 mg QN	-
Day 7, February 10	2 mg QD	500 mg BID	1 mg QN	6 mg BID	-
Day 8, February 11	2 mg QD	500 mg BID	1 mg QN	6 mg BID	Second 20 Hz × 3 s
Day 9, February 12	2 mg QD	500 mg BID	1 mg QN	6 mg BID	Third 20 Hz × 3 s
Day 10, February 13	2 mg QD	500 mg BID	1 mg QN	6 mg BID	-
Day 11, February 14	2 mg QD	500 mg BID	1 mg QN	6 mg BID	Fourth 20 Hz × 3 s
Day 12, February 15	2 mg QD	500 mg BID	1 mg QN	6 mg BID	-
Day 13, February 16	2 mg QD	500 mg BID	1 mg QN	6 mg BID	-
Day 14, February 17	2 mg QD	500 mg BID	1 mg QN	6 mg BID	Fifth 20 Hz × 3 s
Day 15, February 18	2 mg QD	500 mg BID	1 mg QN	6 mg BID	-
Day 16, February 19	2 mg QD	500 mg BID	1 mg QN	6 mg BID	-
Day 17, February 20	2 mg QD	500 mg BID	1 mg QN	6 mg BID	-
Day 18, February 21	2 mg QD	500 mg BID	1 mg QN	6 mg BID	Seventh 20 Hz × 3 s
Discharge instructions	DC	DC	1 mg QN	6 mg BID	DC

## Discussion

The comorbidity mechanism between TD and mood disorders

The patient in this case developed TD following long-term treatment with antipsychotic medications: risperidone (4 mg/day for 11 months) and olanzapine (10 mg/day for 11 months). Symptoms like lip twitching and jaw pain, alongside depression. This clinical progression exemplifies the pattern of drug-induced maladaptive neuroplasticity and comorbidity formation. While antipsychotics exert their therapeutic effects primarily through dopamine D2 receptor blockade, prolonged use induces compensatory neuroadaptive changes. Chronic D2 receptor blockade, particularly within the striatum - a central hub of the basal ganglia - can trigger receptor supersensitivity and subsequent remodeling of corticostriatal circuits, a key mechanism underlying TD [6]. Additionally, abnormalities in the dopaminergic circuit of the basal ganglia-limbic system can worsen depression by affecting reward and emotion regulation [12]. Emotional disorders complicate TD management, often necessitating continued antipsychotic use, which prolongs TD risk [13]. TD-related appearance changes and social impairments can worsen mood disorders through stigma and avoidance [6]. Addressing TD and mood disorders requires both biological and socio-psychological interventions [14].

The potential negative effects of anticholinergic drugs

In this case, the patient had been on long-term benztropine (an anticholinergic agent) at a dose of 6 mg/day for four months prior to admission. Benztropine is commonly used to treat extrapyramidal reactions caused by antipsychotic drugs [15]. However, long-term use of anticholinergic drugs may bring some negative effects, especially in patients with TD. Studies have shown that anticholinergic drugs may lead to cognitive decline (such as inattention and memory impairment) [5,16] and aggravate TD symptoms [17]. After admission, this patient discontinued benztropine, which not only avoided the continuous interference of the cholinergic system but also created conditions for subsequent precise treatment.

The synergistic effect of deutetrabenazine combined with MECT

Deutetrabenazine, a selective VMAT2 inhibitor that reduces the packaging of dopamine into synaptic vesicles, lowers striatal synaptic dopamine concentration and thereby alleviates TD symptoms [6]. In this case, the patient's lip twitching was rapidly relieved after using deutetrabenazine, confirming the core therapeutic value of VMAT2 inhibitors for TD. During the treatment of mood disorders, MECT is also widely used, especially when the drug treatment is ineffective or the patient's condition is severe. MECT can rapidly alleviate severe depressive symptoms and improve the overall mental state of patients [18]. MECT may further enhance the therapeutic effect of deuterobenazine by regulating the neural network and neurotransmitter levels in the brain. Moreover, the combined use of deuterobenazine and MECT may also reduce the side effects of a single treatment method. For example, the mild dyskinesia caused by deuterobenazine and the memory problems caused by MECT may be alleviated in the combined treatment [19]. Theoretically, the combination may mitigate side effects through complementary mechanisms: MECT's enhancement of dopaminergic tone and plasticity could buffer deutetrabenazine's potential over-inhibition of motor circuits, while deutetrabenazine's stabilization of presynaptic dopamine release could buffer MECT's acute disruption of memory networks and aid cognitive recovery.

The implications for clinical practice

This case provides an important reference for the comprehensive management of patients undergoing long-term treatment with antipsychotic drugs. Firstly, a dynamic risk assessment system needs to be established. For patients using drugs with medium to high risk of TD (such as first-generation antipsychotics) and with a treatment duration exceeding 6 months, It is recommended to implement regular screening with the Abnormal Involuntary Movement Scale (AIMS)-a structured clinical tool for detecting and rating TD severity-at least every 6 months for early detection and intervention (American Psychiatric Association, 2020) [20]. and combine the patient's social and psychological status (such as stigma, occupational function) to formulate an individualized monitoring plan. Secondly, the treatment strategy should follow the principle of "precision intervention." When TD coexists with mood disorders, traditional treatment plans that rely on anticholinergic drugs and may exacerbate cognitive or emotional symptoms should be avoided. Instead, targeted VMAT2 inhibitors (such as deuterobenazine) should be selected and may be combined with non-drug methods such as MECT to achieve coordinated symptom relief by regulating dopamine imbalance and promoting neuroplasticity. However, clinicians must weigh these benefits against potential drawbacks: VMAT2 inhibitors can cause sedation, depression, or parkinsonism, and require ongoing monitoring; while MECT, though highly effective, carries risks of transient memory impairment, headaches, and necessitates anesthesia, limiting its accessibility and acceptability for some patients.

## Conclusions

In summary, this case report describes the successful use of combined deutetrabenazine and MECT in a patient with co-occurring TD and recurrent depressive disorder, achieving co-remission of both conditions within a short timeframe. This experience underscores the importance of vigilant monitoring for TD in patients on long-term antipsychotics and suggests that avoiding anticholinergic agents in established TD may be beneficial. Given the limitations inherent to a single-case design and short follow-up, these findings highlight the need for larger, controlled studies to evaluate the efficacy of such combined approaches and to clarify the individual contributions of each intervention.
